# Host-Seeking Behavior and Dispersal of *Triatoma infestans*, a Vector of Chagas Disease, under Semi-field Conditions

**DOI:** 10.1371/journal.pntd.0003433

**Published:** 2015-01-08

**Authors:** Ricardo Castillo-Neyra, Corentin M. Barbu, Renzo Salazar, Katty Borrini, Cesar Naquira, Michael Z. Levy

**Affiliations:** 1 Department of Epidemiology, Johns Hopkins Bloomberg School of Public Health, Baltimore, Maryland, United States of America; 2 Center for Clinical Epidemiology & Biostatistics - Department of Biostatistics & Epidemiology, University of Pennsylvania Perelman School of Medicine, Philadelphia, Pennsylvania, United States of America; 3 School of Science and Philosophy, Universidad Peruana Cayetano Heredia, Lima, Peru; Universidad Autónoma de Yucatán, Mexico

## Abstract

Chagas disease affects millions of people in Latin America. The control of this vector-borne disease focuses on halting transmission by reducing or eliminating insect vector populations. Most transmission of *Trypanosoma cruzi*, the causative agent of Chagas disease, involves insects living within or very close to households and feeding mostly on domestic animals. As animal hosts can be intermittently present it is important to understand how host availability can modify transmission risk to humans and to characterize the host-seeking dispersal of triatomine vectors on a very fine scale. We used a semi-field system with motion-detection cameras to characterize the dispersal of *Triatoma infestans*, and compare the behavior of vector populations in the constant presence of hosts (guinea pigs), and after the removal of the hosts. The emigration rate – net insect population decline in original refuge – following host removal was on average 19.7% of insects per 10 days compared to 10.2% in constant host populations (p = 0.029). However, dispersal of *T. infestans* occurred in both directions, towards and away from the initial location of the hosts. The majority of insects that moved towards the original location of guinea pigs remained there for 4 weeks. Oviposition and mortality were observed and analyzed in the context of insect dispersal, but only mortality was higher in the group where animal hosts were removed (p-value <0.01). We discuss different survival strategies associated with the observed behavior and its implications for vector control. Removing domestic animals in infested areas increases vector dispersal from the first day of host removal. The implications of these patterns of vector dispersal in a field setting are not yet known but could result in movement towards human rooms.

## Author Summary

Chagas disease is transmitted by triatomine bugs that actively disperse by walking and flying. The control of this vector-borne disease focuses on reducing or eliminating the insect vector populations. Most transmission of *Trypanosoma cruzi*, the causative agent of Chagas disease, involves insects living within or very close to households and feeding mostly on domestic animals. As animal hosts can be removed due to migration, slaughter, or death, it is important to understand how host availability can modify transmission risk to humans and to characterize the dispersal of triatomine vectors on a very fine scale. We used a semi-field system to characterize the dispersal of *Triatoma infestans*, and compare the behavior of vector populations in the constant presence of hosts and after the removal of the hosts. The emigration rate – net insect population decline in original refuges – following host removal was on average 19.7% of insects per 10 days compared to 10.2% in constant host populations. Activity of insects was significantly increased when hosts were removed. The removal of domestic animals in infested areas increases vector dispersal, possibly towards nearby human sleeping spaces.

## Introduction

Chagas disease, a vector-borne disease caused by the parasite *Trypanosoma cruzi*, affects from 7 to 8 million people in the Americas [1]. The vast majority of people infected are not detected [2], [3], and when the disease manifests clinically it cannot be reversed and can be fatal [2]–[4]. In addition to the inability to detect early cases and the lack of treatment in advanced stages, currently there are no vaccines available to prevent infection [5]. Therefore, efforts to halt transmission are crucial to reduce the burden of disease [6].

Most control programs aimed at halting *T. cruzi* transmission focus on reducing the insect vector populations [6]. In the Southern Cone of South America, transmission of *T. cruzi* is mostly driven by *Triatoma infestans* living within or very close to households, at times feeding on humans, but feeding mostly on domestic animals [7], [8]. The proximity between animal corrals and human bedrooms, especially in urban areas or densely populated rural areas, may facilitate dispersal of vectors from animal enclosures into human houses.

Several studies have reported the presence of guinea pigs in houses as a risk factor for triatomine infestation in endemic areas [1], [9]–[11]. In Arequipa, Peru, an area where Chagas disease is an emerging and re-emerging problem, the presence of domestic guinea pigs increases the odds of triatomine-insect infestation by 1.69 times and the triatomine density by 2.4 times [2], [3], [12]. In this setting, guinea pigs are raised in small numbers in backyards, on rooftops, and inside houses. Guinea pigs are an important source of protein in the region. Many reasons can lead to changes in the distribution and presence of domestic animals of different species. Because guinea pigs are typically fed with alfalfa, the price of which fluctuates widely [2]–[4], [13], and because of their small number in a corral, guinea pigs are often withdrawn during certain parts of the year and the corral left empty. The loss of hosts is presumably a catastrophic event for local triatomine populations relying on those animals as a source of blood meals. In other areas where the animal species composition differs, animals could be withdrawn from corrals due to death, migration, trading, or slaughter, and these events might pose the same threat for triatomine populations. When hosts are removed from corrals the triatomine insects that live, reproduce and feed on them might either leave, or stay to wait for a new wave of hosts. If the triatomine vectors disperse from their refuge in search of new hosts, the removal of animal hosts likely implies a sudden and important rise of the risk for the human populations.

The dispersal behavior of different triatomine vectors has been studied from various perspectives. *T. infestans* uses two types of locomotion for dispersal: flying and walking. In Argentina most *T. infestans* were found walking in infested areas, but a number were captured flying [5], [14]. To add complexity to the locomotion patterns observed in *T. infestans*, it has been reported that the initiation time of flight shows wide variability based on climatic and individual factors [6], [15] and that *T. infestans* do not fly above 2,750 m [6], [16], an important feature in the highly populated cities of the Andes with altitude in this range such as Arequipa in Peru and Cochabamba in Bolivia. For some triatomine species that use flight as an important type of locomotion, such as *Triatoma dimidiata* [7], [8], [17], [18], light is a physical cue that might attract insect into houses [19], and streetlights have been associated with increase domestic infestation [20]. Some studies have reported cues for walking dispersal related to host seeking, mainly by isolating the effect of a chemical cue. Taneja and Guerin [21] noted that carbon dioxide is an important cue for host location by triatomine insects; however, its attractant effect was not stronger than host odor. Barrozo and Lazzari [22] found that *T. infestans* moves towards airstreams enriched with CO2 and the accuracy of the orientation increases with CO2 intensity. They also found that L-lactic acid did not show an effect by itself, but its combination with CO2 had a synergistic effect that increased a positive orientation. Taneja and Guerin also found in 1997 that urine and its component ammonia attract triatomines [23]. The joint attractive effect of the volatile compounds of growing yeast was demonstrated under laboratory conditions by Guerenstein et al. [24] and under field conditions by Lorenzo et al. [25]. Guerenstein and Guerin [26] found that there is an activation effect caused by nonanal and that isobutyric acid increases triatomine upwind displacement. Another source of chemical compounds that causes triatomine aggregation are the triatomines themselves. Schofield and Patterson [27] found that triatomine feces contain an assembly pheromone that attracts unfed larvae and stops the locomotion of fed larvae. Lorenzo et. al. [28] demonstrated that nymphs of *T. infestans* tend to aggregate around papers impregnated with dry feces, but not papers with fresh feces. Lorenzo and Lazzari [29] found that *T. infestans* aggregates not only around papers impregnated with its own feces, but also with feces of *T. guasayana* and *T. sordida*. Also Lorenzo and Lazzari [30] reported a response of *T. infestans* to chemical footprints left by walking insects. Experimentally they showed that the cuticle plays a role in this signaling process. Interestingly, Reiseman et al. [31] described a differential response to feces based on color lights.

Other studies have reported the role of physical cues on host seeking by triatomines. Lazzari and Nuñez [32] and Flores and Lazzari [33] reported the importance of heat from warm-blooded animals on the displacement of triatomines towards food. Barrozo et al. [34] described the role of water vapor, a common and constant by-product of animal respiration, on the orientation of triatomines. Finally, Catala et al. [35] proposed a hypothesis about the role of infra-red radiation on the dispersal of *T. infestans.* The current knowledge about triatomine behavior in relation to different attractants has led to the development of traps baited with host-based cues, light traps, and artificial shelters [36]. All these studies, and their applications, analyzed displacement of triatomines towards chemical and physical cues; however, it is not yet known how triatomine insects disperse in the event that their hosts, the sources of all these cues, completely disappear from their surroundings.

In order to determine how the vectors behave under such circumstances, we characterized the initial dispersal behavior of triatomine insects in a small-scale area when the only source of blood meals is withdrawn from the environment, and compared it to an identical environment in which the host population remains constant. The main objective of our study was to test the hypothesis that *T. infestans* migrate at a faster rate when their hosts are removed compared to *T. infestans* in continuous presence of hosts.

## Methods

### Ethical statement

The Institutional Animal Care and Use Committee (IACUC) of Universidad Peruana Cayetano Heredia reviewed and approved the animal-handling protocol used for this study (identification number 60942). The IACUC of Universidad Peruana Cayetano Heredia is registered in the National Institutes of Health at the United States of America with PHS Approved Animal Welfare Assurance Number A5146-01 and adheres to the Animal Welfare Act of 1990.

### Experimental design

**Figure 1:**
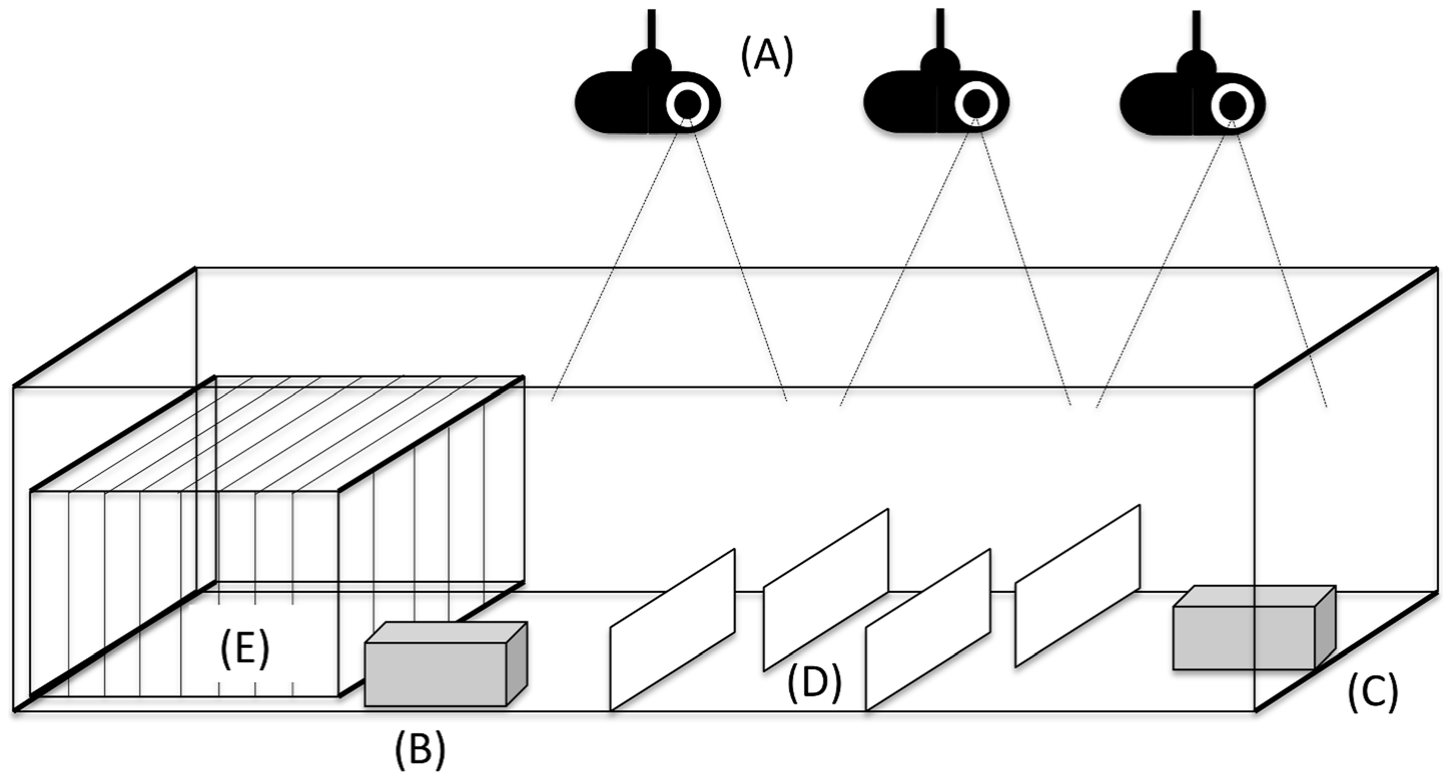
Diagram (not to scale) of experimental design for each glass tank. Video cameras recording each area of the tank (A); primary (B) and secondary (C) refuges; glass-walled maze (D); and guinea pig cage (E). Green dots represent triatomine insects in the primary refuge (R1); red dots represent triatomine insects in the secondary refuge (R2); black dots represent triatomine insects in or under the guinea pig cage (Q1); and grey dots represent no triatomine insects. Q2, Q3, and Q4 stand for quadrants 2, 3, and 4.

In order to understand the dispersal of *T. infestans* after bloodmeal sources are removed, we conducted an experiment in a semi-field system with motion-detection cameras. In two 10-foot long glass tanks with a glass-walled maze in the middle area and floor covered with 2-foot x 3-foot sheets of white paper, we placed a cage with two guinea pigs at one extreme of the tanks. A cardboard box with corrugated paper to increase internal surface (*the primary refuge*) containing 60 triatomine insects was placed in each tank proximal to the guinea pig cage. An identical carboard box (*the secondary refuge*) was placed at the extreme of the tank opposite to the guinea pig cage and the primary refuge (Fig. 1). After a week of cohabitation, the guinea pigs from one of the tanks were removed (*time of intervention*); this tank was designated the *intervention tank* and the tank with constant presence of guinea pigs was designated the *control tank*. All triatomine insects were fasted for 2 weeks before cohabitation with guinea pigs started, and insects had free access to the hosts during the time hosts remained in the tanks.

We defined six discrete locations across each experimental tank: quadrants 1–4 and the primary and secondary refuges. Quadrant 1 refers to the area occupied by the guinea pig cage at one extreme of the tank; the primary refuge is proximal to this quadrant. Quadrant 2 refers to the area immediately surrounding the primary refuge and the beginning of the maze. Quadrant 3 refers to the middle portion of the maze. Quadrant 4 refers to the final portion of the maze and the area immediately surrounding the secondary refuge, which was placed at the extreme of the tank (Fig. 1). The insects were free to move throughout all areas of the tank.

During the experimental period, we counted the number of *T. infestans* in these six different areas of the intervention and control tanks, video-recorded their movements, and estimated their level of activity by motion-activated snapshots. The experiment was repeated three times. We used a data logger to record temperature and relative humidity every 10 minutes during the three replicates of the experiment.

### Animals

We used a total of 12 one-month-old female guinea pigs from a local farm free of triatomine infestation and a total of 360 triatomine insects that were raised in a large triatomine colony originated from Arequipa, Peru. In order to form the 60-triatomine groups for each tank and each repetition, we chose 10 triatomine insects from the following six groups: 2nd, 3rd, 4th, 5th instar, female and male.

### Data collection

We used a video camera system to observe and record two types of files; snapshots of each quadrant of each tank every 10 seconds and snapshots of a quadrant where any movement was detected to a maximum of two motion-activated snapshots per second. Because of the continuous movement of guinea pigs in quadrant 1, we only used snapshots from the other 3 quadrants where activation of snapshots was solely related to triatomine movement. Because triatomines are nocturnal, we used red-light bulbs between dusk and dawn to provide illumination for the cameras. To confirm the lack of disturbance of red light in the dispersal of *T. infestans* we conducted a pilot with three different light colors: white, green, and red. We illuminated a 2-square-foot-area tank containing 20 *T. infestans* and observed their behavior. Under white and green light the triatomine insects stayed on the edge of the tank and only moved along the borders, a behavior consistent with negative phototaxis. Under red light the triatomine insects moved all over the tank surface without showing any pattern that would suggest light disturbance (S1, S2, and S3 Figs.).

We also visually examined each tank at around 10 AM every three days and the day before and after removing guinea pigs. We counted the number of triatomine insects in each quadrant and box and registered their sex or developmental stage and their nutritional status. The nutritional status was only recorded during the 7 first days of cohabitation prior to intervention. The nutritional status was measured in a scale from 1 to 4, and was based on a qualitative determination of blood reserves in the midgut developed by Montenegro [37].

During these in-person observations, we recorded the number of dead insects and eggs laid in each quadrant or refuge. We withdrew the eggs from the tanks upon observation, but left dead insects where they were found.

### Statistical analyses

#### Analysis of videos

We compared the activity of triatomine insects during the observation periods by analyzing the daily total number of motion-activated snapshots per tank taken by the cameras. We used a hierarchical linear model with random intercept to model the average number of motion-activated snapshots depending on the intervention. We used the recorded temperature and relative humidity every 10 minutes to calculate the median daily temperature and the median daily relative humidity to include them as regressors.

#### Analysis of counts of insects

To model the differential dispersal of triatomine insects in the intervention and control tanks we used a Poisson regression on the insect counts in the primary refuge over time depending on the presence or absence of hosts. We first considered a simple Poisson regression. Second, we considered a hierarchical Poisson regression with random intercept to account for different counts in the refuge at the time of intervention. Finally, to allow random variation of the rate of emigration, or insect count decline in the primary refuge, between repetitions, we used a hierarchical Poisson regression with random intercept and slope. We also included as regressors the median daily temperature and the median daily relative humidity. The fit of the alternative models to the data was compared with Akaike's Information Criterion (AIC).

We plotted the observed number of triatomine insects in each quadrant and boxes over time. We also plotted the observed number of triatomine insects in the box close to the guinea pig cage as a function of time and the predicted number obtained from the hierarchical Poisson regression with random intercept, the model with lowest AIC. The number of triatomine insects that were found in the original insect refuge the day before the intervention was compared with chi-squared test.

#### Death and oviposition

Finally, we evaluated the number of eggs found over the study area and compared the number of dead insects between intervention and control tanks with a log-binomial regression. All graphs and analyses were produced in R [38] and all estimates were analyzed at α level  = 0.05.

## Results

**Figure 2:**
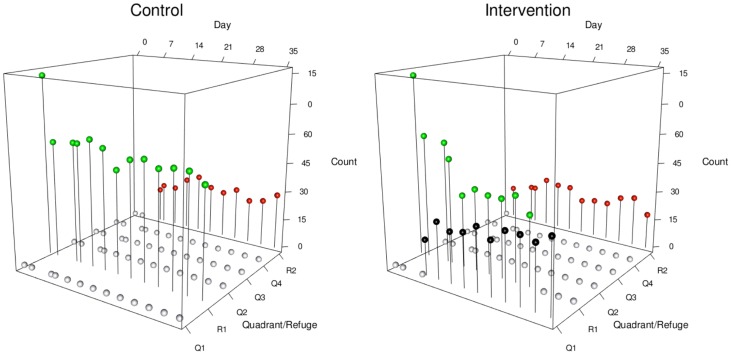
Distribution of triatomine insects on tanks over time in one of the three repetitions.

**Figure 3:**
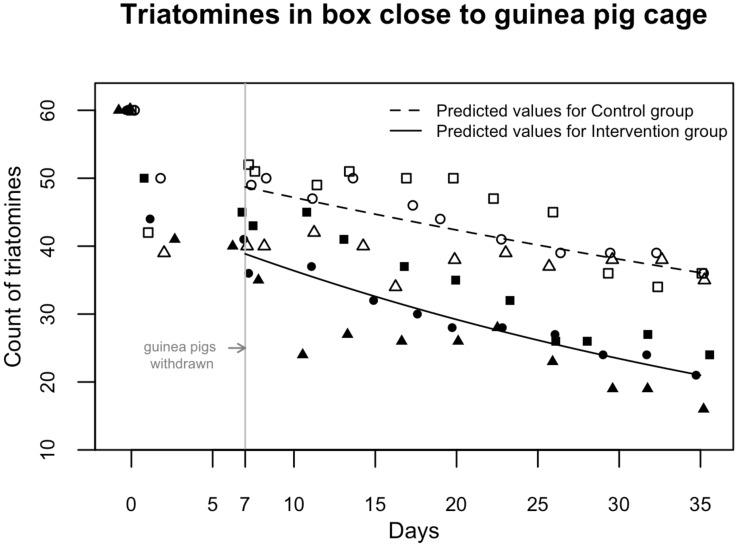
Observed and predicted number of triatomine insects as a function of time. White symbols represent control group and black symbols represent intervention group. Symbol shapes differentiate between repetitions.

Across the three repetitions, we observed a similar pattern of emigration of insects; the number of triatomine insects in the primary refuge decreased faster in the intervention tank than in the control tank (Figs. 2 and 3). We also observed that triatomine insects in the intervention tank leaving their primary refuge dispersed in both directions, towards and away from the empty guinea pig cage (Fig. 2). In the control tank the number of triatomine insects in the primary refuge reduced slightly over time and those triatomines that emigrated consistently did so towards the secondary refuge. Fig. 2 illustrates the dispersal data over time from one of the repetitions, although we observed the same pattern in all three repetitions. In this figure, the number of triatomine insects that were found in their primary refuge is represented by green dots, and insect emigration from the primary refuge is represented by the reduction of those dots over time. This reduction is more marked in the intervention tank. Emigration from the primary refuge to the secondary refuge was prevalent in both tanks and is represented by the red dots. However, in the intervention tank alone, some insects left their primary refuge and dispersed towards the empty guinea pig cage; this emigration is represented by the black dots.

**Figure 4:**
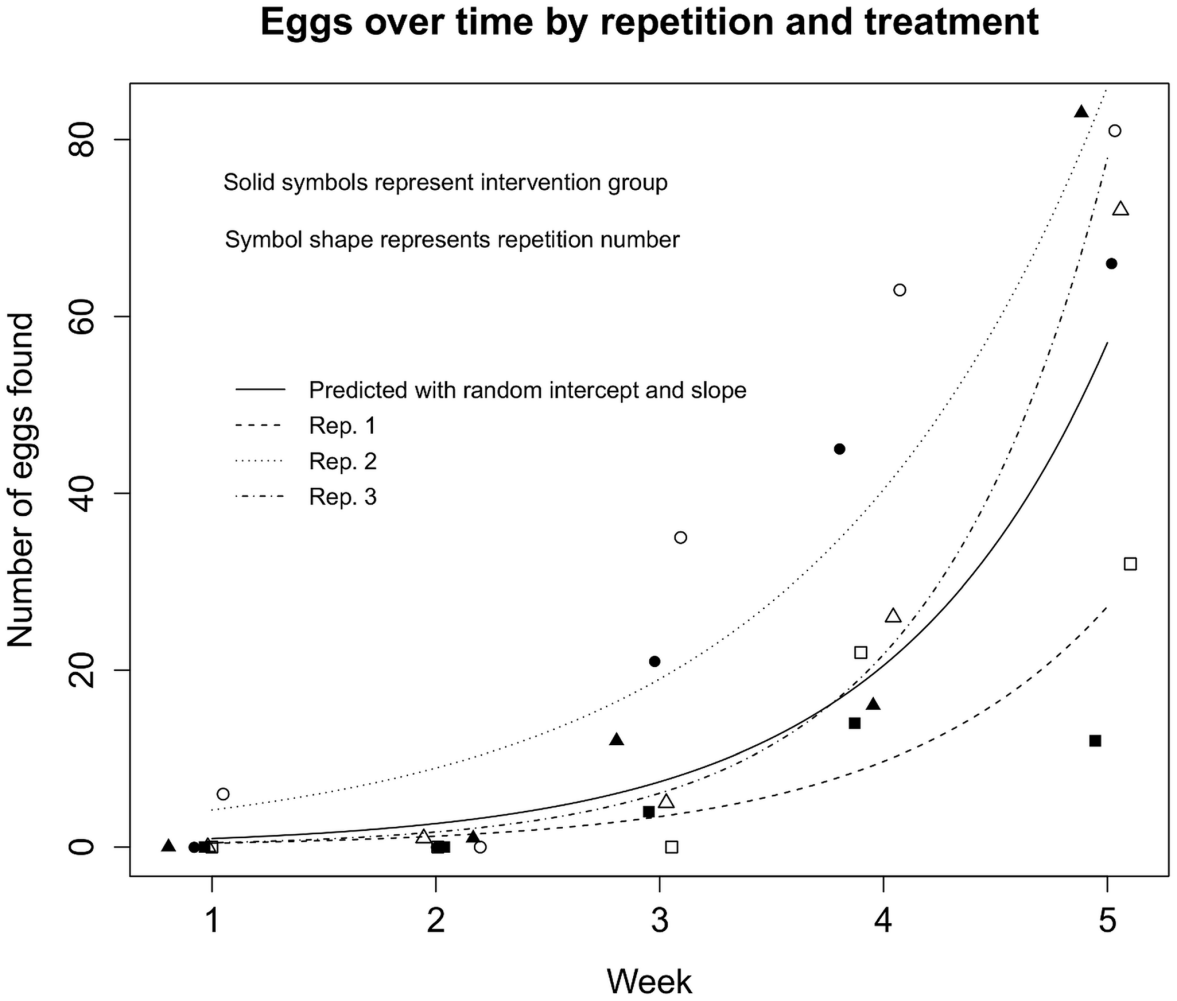
Observed and predicted number eggs as a function of time. Predictions based on outputs from Poisson model with random intercept and random slope.

When we evaluated dispersal by developmental stage and sex, we observed more complex patterns. The most remarkable finding is that females, independently of the tank, started dispersal immediately after they were placed in the experimental area. A proportion of the females quickly found the secondary refuges and stayed there. On average, 32% of females were observed in the secondary refuge the day before intervention. A related observation was the presence of eggs in the primary and secondary refuges and across the quadrants. We did not observe any pattern in the distribution of eggs over the study area, but the number of laid eggs increased exponentially over time in the three repetitions in both tanks as shown in Fig. 4.

The three Poisson regression models we used to quantify the rate of emigration of *T. infestans* from their primary refuge in our system estimated similar rates, with an average of 10.2% and 19.7% reduction of insects in the primary refuge per 10 days lapsed in the control group and the intervention group, respectively. This difference was statistically significant, with p-values between 0.029 and 0.036 depending on the model (Table 1). Overall, the AIC favored the hierarchical Poisson model with a random intercept (Table 1), suggesting that the counts of insects in the refuge at the time of intervention might be different between repetitions but the net emigration rates after the intervention are consistent across repetitions. Nevertheless, the heterogeneity in the number of triatomine insects in the primary insect refuge at the time of intervention was not statistically significant (chi-squared  = 0.3748; df  = 2; p-value  = 0.83).

**Table 1:** Comparison of Poisson regression models to assess the emigration rate.

	Poisson model			Poisson with random intercept			Poisson with random intercept and slope		
	Coef.	SE	p	Coef.	SE	p	Coef.	SE	p
Intercept	3.964	0.073	<0.001	3.961	0.089	<0.001	3.956	0.114	<0.001
Tank	−0.147	0.113	0.195	−0.147	0.113	0.195	−0.150	0.113	0.187
Time (days)	−0.011	0.003	0.001	−0.011	0.003	0.001	−0.010	0.004	0.011
Tank*Time	−0.011	0.005	0.029	−0.011	0.005	0.029	−0.011	0.005	0.036
*AIC*	*361.85*			***31.28***			*45.76*		

**Figure 5:**
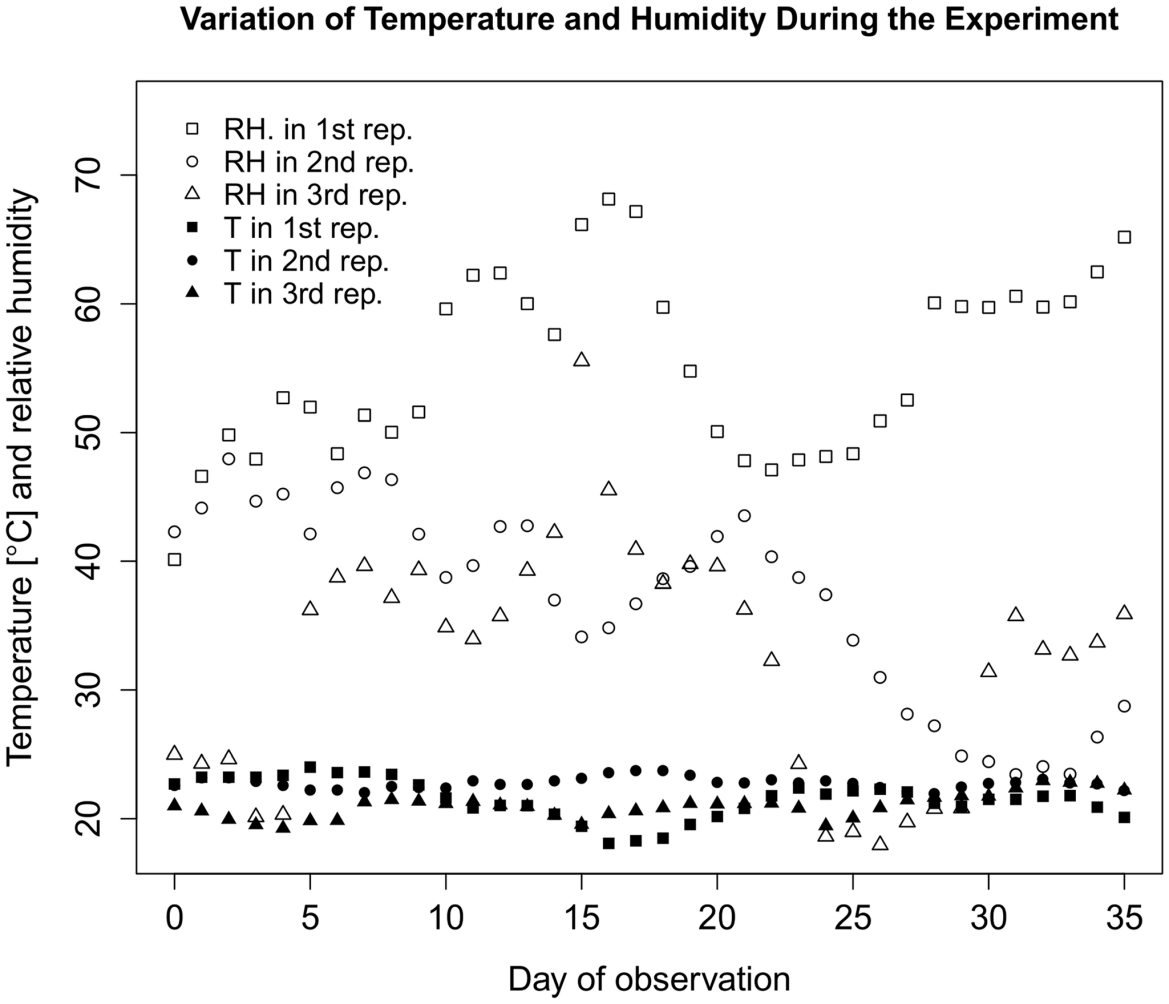
Median daily temperature and relative humidity across repetitions.

The dispersion of the data around the values predicted by the best model is presented in Fig. 3. The nutritional status of triatomines found in the secondary refuge the day before intervention was not statistically different when we compared the intervention versus the control tank (Fisher's exact test p-value = 0.39). We observed high variability of daily median relative humidity and low variability of daily median temperature as observed in Fig. 5. These weather variables had neither an important effect size nor a statistically significant influence on the emigration rate.

**Figure 6:**
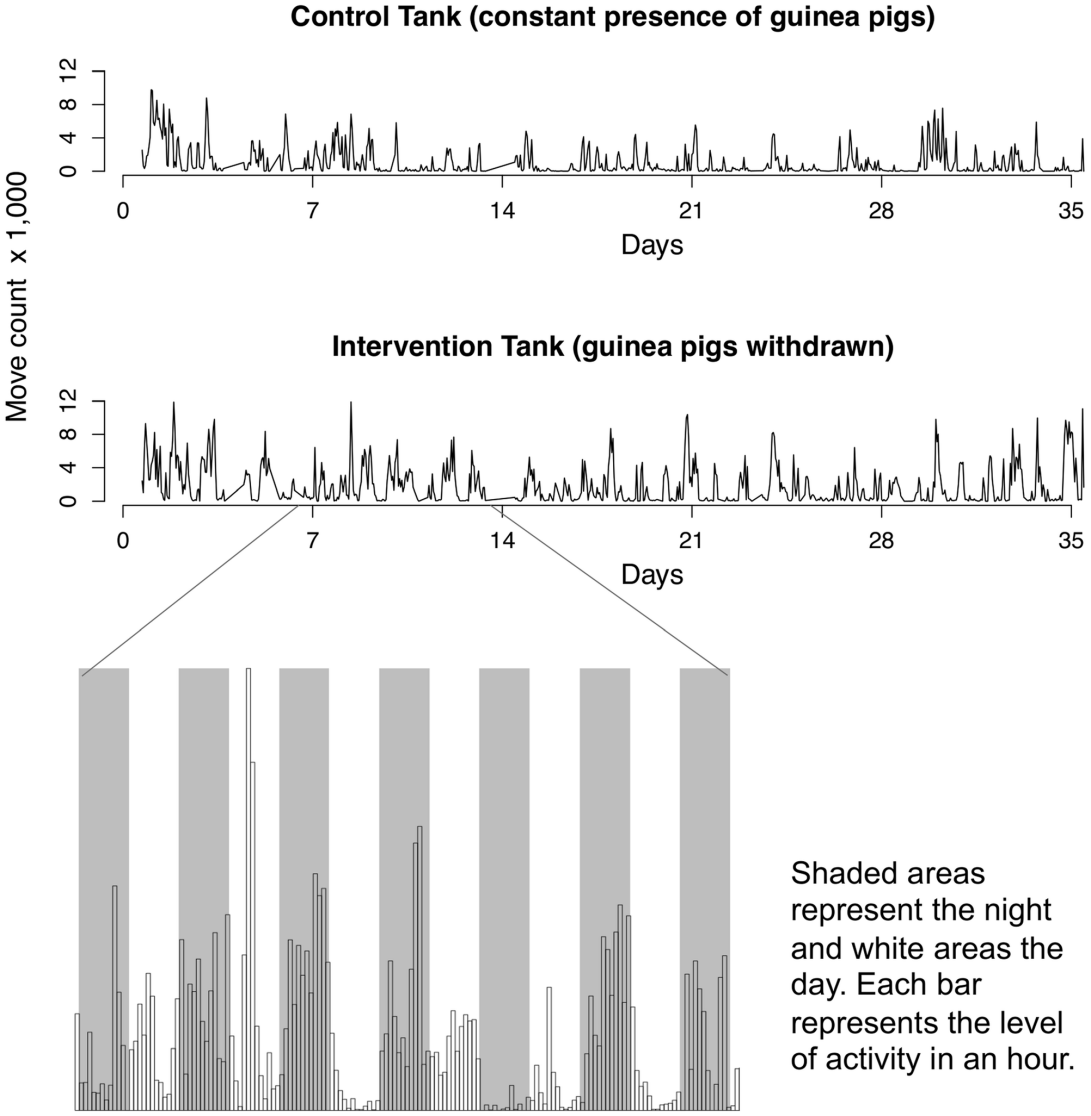
Activity level estimated by count of motion-activated snapshots. Graph shows count of moves recorded in one repetition. The inset shows a detailed histogram of moves during days and nights.

In terms of observed level of insect activity, there were complex patterns in the intervention tank after guinea pigs were removed. The night after the guinea pigs were removed, we observed an elevated number of movements recorded by our system in the intervention tank compared to the control tank. S1 Video shows quadrant 1 and primary refuge of the intervention and control tanks between 11 PM and 2 AM the night after guinea pigs were removed. The video was created with the snapshots taken automatically every 10 seconds and clearly shows the high level of activity in the intervention tank. Over the observation period, the level of activity was higher in the intervention tank, and can be observed by comparing the spikes in Fig. 6. The average number of motion-activated snapshots per day was higher in the intervention tank compared to the control tank by 11,186 snapshots across all three repetitions (95% CI: 4,653–17,720; p = 0.001). A one Celsius degree increase in the median daily temperature was associated with an increase of 5,068 motion-activated snapshots per day (95% CI: 1,317, 8,818; p-value = 0.01), and an increment of one percentage point of relative humidity was associated with an increase of 302 motion-activated snapshots per day (95% CI: −49, 655; p-value = 0.10). An observation related to the frequency of movements recorded by our system is that insect activity started on average 1 hour and 12 minutes earlier in the intervention tank compared to the control tank the night after hosts removal.

The probability of triatomine insects dying in the intervention group was 1.46 times higher than in the control group and this increase was statistically significant (95% CI: 1.10, 1.95; p-value = 0.0098). Overall, most dead triatomine insects were found in the primary refuge. In total, in the three repetitions, 24 out of 33 dead insects were found in the primary refuge of the control tank, and 34 out of 52 dead insects were found in the primary refuge of the intervention tank.

## Discussion

Here we characterize the ex-situ dispersal of *T. infestans* after removal of sources of blood. The observed reduction of insect count in their primary refuge was, on average, 19.7% over 10 days, and triatomine insects did not distribute randomly over the available area. Some of the insects remained in the primary refuge (close to the guinea pig cage), some migrated to the secondary refuge (far from the guinea pig cage), and some dispersed towards the empty guinea pig cage. The empty guinea pig cage was the only source of olfactory cues associated with blood sources, and also offered shelter to bugs.

In r/K selection theory some species develop an r strategy that favors population growth rate (r) with low intra-species competition, and many offspring with low probabilities of survival, while others develop a K strategy, where population growth is limited by carrying capacity of their environment (K), few offspring are produced, all with a high probability of survival, and intra-species competition is high [39]. These two strategies also correspond to different dispersal patterns: a pure r-strategy would involve a continuous high level of dispersal that would minimize the impact of perturbations, and conversely, a pure k-strategy would minimize dispersal under constant conditions, but may display significant dispersal in the face of perturbation [40]. Active dispersal of triatomine insects has been mainly associated with seeking food and mating [41]; the r-strategy may also serve to find and colonize new areas, maximizing the overall survival of the descendants in the case of unpredictable environments [42]. Rabinovich proposed that *T. infestans* should be considered a K-strategist based on population growth, average longevity, starvation resistance, and dispersal capacity [43]. However, he also recognized that little was known about the dispersal capacity of *T. infestans*. The significant dispersal we observed to the refuge at the other side of the tank, even with blood sources at immediate proximity suggests that part of the dispersal of *T. infestans* is linked to an r-strategy type dispersal, while the significant impact of the removal of the hosts on the dispersal rate confirms that there is a K-strategy type dispersal component for a mixed strategy in *T. infestans* dispersal.

We observed two opposite responses after hosts were withdrawn; a proportion of insects stayed close to the host cage, while others migrated away. One explanation for the prolonged presence of triatomines close to the empty host cage is the continued presence of chemical attractants for triatomines. Specifically, urine [21], [23], humid feces, or even humid scraps of pasture greens may attract triatomines [34]. Remaining near a previously present food source may also be an advantageous strategy in areas where foraging for new food sources carries a high cost. Triatomine insects have a number of nocturnal predators such as geckos [44], rodents [45], and spiders [46], and diurnal predators such as chickens [47], dogs [48], and cats [48]. In addition to the risks of predation, seeking new food sources might expose triatomine insects to desiccation, as occurs with other hemiptera insects [49], and possibly deplete the insects' energy stores, as suggested by the observations of Abraham et al. [50] who found that flying *T. infestans* had a poorer nutritional status than those captured close to animal corrals. Interestingly, this potential strategy associated with a passive food-seeking behavior (waiting in a high-probability-of-food zone) could explain some previous field observations by our team. In 4 rural communities of Arequipa, Peru, we conducted entomological surveys for triatomines in each animal corral in the area. Among 1762 animal corrals we found *T. infestans* in 294, and 104 of these infested corrals did not contain any animals. Those empty corrals might represent high-probability-of-food zones that are exploited by triatomine insects and should be targeted in vector-control strategies. The opposite response observed in our experiment, a large proportion of insects migrating away from the empty cages, may be associated with an active-food-seeking strategy, useful when the risks outside of the refuge are lower than the risk of starving by remaining in the refuge.

The increased frequency of movements and the earlier start of insect activity observed in the intervention tank versus the control tank may be explained by active search for a host. This active search most likely was performed by unfed triatomines, while engorged triatomines could stay or avoid being in the proximity of hosts. Surprisingly, across the three repetitions this differential behavior started the same night that guinea pigs were withdrawn. Based on our pilot observations and the biting rate range (0.29 to 0.59) reported by Lopez et al. [51], we left the insects from both tanks to cohabitate with the guinea pig for one week prior to withdrawing the hosts from the intervention tank, and it is possible that most insects had fed on the host early during that week, and would have needed to feed again the same night of host removal. Hosts might also be sources of heat for proper enzymatic activity and be sought by engorged or partially full triatomine insects to facilitate digestion [52].

Previous studies have examined a number of determinants of triatomine dispersal. The presence of hosts for blood meals has been linked directly to the nutritional status of triatomine vectors and to their population density [53], [54]. Nutritional status can affect female triatomine fecundity, but its main impact on population density is by modifying the duration of the egg-to-adult development period [53]; therefore, the effects of nutritional status on triatomine population density and dispersal would only be seen over several months. In our 5-week-long repetitions, we examined the immediate and short-term effects on triatomine dispersal caused by sudden host removal, a common event in infested areas of Arequipa, Peru. Several studies have reported the association between nutritional status of triatomine vectors and flight dispersal or flight potential [55]–[58] and Ramsey and Schofield even discussed the risk associated with passive transportation of passive triatomines [59]. The role of walking triatomines and its relationship with triatomine nutritional status has been suggested [50], [60]. In *Rhodnius prolixus*, a model of triatomine physiology, nutritional status has been found to have an effect on sensory response to host cues, and this is likely to partially explain the host-seeking behavior observed in our system. In the intervention tanks the only host-related cues remained in the empty corral, which may have drawn triatomines to them as their nutritional status decreased over the duration of the trial. However, triatomines dispersing beyond the empty corral might be explained by exploration of the unknown when blood meals are required and not found upon following host-related cues.

We observed that in both tanks, control and intervention, triatomines were usually found in the extremes of the tanks during in-person observations (∼10 AM). Ideal free distribution theory [61] proposes that animals know the quality of the patches (distribution of resources) where they move and will choose patches with higher quality. In our intervention tank, after removing guinea pigs, the quality of the quadrants in terms of food became the same; however, the presence of the refuges as well as the presence of the empty guinea-pig cage with feces, urine and scrapes of alfalfa provided cues as well as safe harbor for insects, making some quadrants more attractive than others. Thus, ideal free distribution might explain the similar distribution of *T. infestans* in areas far from and close to the location of blood-meal sources after removal of hosts.

We found *T. infestans* eggs scattered across the experimental tanks, without any clear pattern. The absence of spatial pattern in the distribution of eggs might suggest a strategy to disperse eggs around the original colony, and could support the null hypothesis of the ideal free distribution of triatomine insect females over the experimental area. It is also possible that triatomine insects did not find an appropriate substrate to lay their eggs [62]. The importance of walking pregnant triatomine females was reported by Abrahan et al. in 2001 [50], who suggested that this type of locomotion in females is an adaptive strategy that allows for dispersal of many eggs. Dispersing eggs around the original colony increases the chances of at least one egg surviving and it is a preferred strategy as the intensity of predation increases [63]. Egg dispersal might also increase colonization success and help to avoid reaching carrying capacity [64] if the colony only grows with a limited supply of blood meals or nesting space.

We did our best to maintain the intervention and control groups under identical conditions throughout the experiments. Despite our efforts, there were some insects that initiated dispersal in the intervention tank before guinea pigs were withdrawn in two of the three repetitions. The slight difference in the number of insects in the original refuge before the guinea pigs were withdrawn was not statistically significant for any of the three repetitions. For all repetitions the control and intervention tanks were switched, and in all cases the insects started the repetitions in a completely clean tank without residues from previous experiments that could have leaved traces of olfactory cues. One potential explanation we propose is that triatomine insects display a clear r-strategy, dispersing considerably even when they have reliable food sources and refuge [65], [66].

We faced some limitations that should be taken into account when making inferences from our results to *in-situ* triatomine dispersal. In our system the substrate of the tanks' floor was white paper and within the refuges was corrugated cardboard. In the wild, triatomine insects are exposed to a wide variety of materials which can alter their physiology and behavior [67] and their fecundity [62]. We had a fixed number of triatomines by developmental stage and sex which did not reflect the stable population distribution of *T. infestans* [43]. Patterns of dispersal, however, might be influenced by density and stage structure of vector colonies [68]. We kept a fixed number of guinea pigs across all repetitions, but the ratio of hosts to vectors might also influence dispersal patterns [69]. Also, the number of triatomine insects per tank reduced slightly during the experiment due to mortality. In field conditions the population might recover or increase through reproduction, especially in corrals with a constant source of blood meals for the production of eggs. Changes in population size might affect dispersal, especially when the number of insects exceeds the carrying capacity of the environment [70], [71]. In addition, we observed our *ex-situ* system for only 28 days after the removal of the hosts. The dispersal of triatomines would certainly have continued past our period of observation, and different patterns could have emerged over longer time scales. We observed a smooth decrease of triatomines in the primary refuge over 4 weeks. If it were the case that chemical cues associated with the empty guinea pig cage did not attract triatomines following that period, a sudden dispersal of triatomines would be observed. This effect could be accentuated by the increasing presence of feces associated to dispersing triatomines in the secondary refuge, since triatomine feces and triatomine aggregation are strongly associated [27]–[29]. In our system there was significant variation in relative humidity and small variation in temperature over time. A greater variability of temperature and relative humidity would be expected in field conditions across different ecotopes and in peridomestic areas, probably influencing the activity and the dispersal of *T. infestans*, in keeping with our observed increased activity associated with higher temperature. Also, we purposely did not place attractants in the other side of the tanks such as secondary sources of bloodmeals in an effort to mimic situations in which migration would entail seeking an entirely new food source. In areas where animal corrals are close to one another or to rooms where humans sleep most migration might be directed to such an area. Cues from proximal animals or humans would be detected soon after food seeking starts and then traces of those cues would lead the insects directly to the hosts [72]. It is likely then that the observed propensity to disperse when hosts are removed would be even higher in a field environment. Finally, our experimental design included three replicates of the experimental units to avoid the effect of stochasticity, as suggested by Hurlbert [73]. Hurlbert also discusses other study design tools to avoid the problem of pseudoreplication. He emphasizes the need of controls, randomization, independence and interspersion [73]. We applied all those tools in the design and statistical analyses, but there is still potential for dependence of the studied emigration pattern in each tank. As described by Lorenzo and Lazzari[30], walking triatomines leave traces on the floor, orienting other triatomines in their dispersal. Even though we could not prevent this effect, and the observation over time in each tank might be not be independent, we think our observations “still contain useful information…”, as Hulbert states about some imperfect studies [73].

Our results represent triatomine dispersal in areas of low-animal-corral density, where chemical and physical cues associated with the presence of animals are sparse and diluted, and might, as well, represent initial dispersal of triatomine insects in areas of high-animal-corral density. While dispersal is important even in the constant presence of hosts, there is an important change in the dispersal pattern when hosts are removed. We observed two types of dispersal: close spatial association with original location of removed hosts, and dispersal, seemingly at random, far from the primary refuges and host locations. Our study was not set up to conclude if any ecological or evolutionary theory explains the observed patterns of dispersal; further studies would help to determine the ecological and evolutionary meaning of these dispersal strategies, and could answer if behavioral patterns are the result of bet hedging [74] and/or random individual variation [75]. Also, we focused our study in the interaction between host and vector. The presence of the parasite *T. cruzi* might change the host-vector interaction; more complex experiments considering the parasite, host and vector are needed to assess this possibility. We used guinea pigs for our experiments, but our results may extend beyond that species. In other areas goats [76], dogs [8], and poultry [77], [78] have been described as host highly associated with the presence of triatomines. Our observed effect of host removal on triatomine dispersal may change depending on the population dynamics of domestic species. Depending on the rate of removal and replacing of hosts, a higher proportion of continuous triatomine dispersal could be observed without sudden increases in insect activity, or on the contrary, questing in corrals that would shortly be repopulated could be more common. The removal of these animal hosts may lead to sudden infestations of surrounding areas by insects looking for other sources of blood meals, increasing the risk of *T. cruzi* transmission for humans in proximal areas. Further studies are needed to discern adequate strategies to limit *T. infestans* dispersal in these settings and the associated increase of transmission risk. Additionally, empty animal corrals may remain attractive to the vectors or be used by triatomines as hiding places and should be carefully considered in vector-control activities such as monitoring, insecticide treatment, and housing improvement [12], [79]–[81].

## Supporting Information

S1 Video Video created from snapshots taken automatically every 10 seconds between 11 PM and 2 AM the night after guinea pigs were removed. The covered area includes quadrant 1 and primary refuge of the intervention and control tanks.
(MOV)

S1 Fig In our pilot study triatomine insects under white light tend to stay on the borders of the tank and only move along the borders, a behavior consistent with negative phototaxis.
(TIF)

S2 Fig In our pilot study triatomine insects under green light tend to stay on the borders of the tank and only move along the borders, a behavior consistent with negative phototaxis.
(TIF)

S3 Fig In our pilot study triatomine insects under red light move all over the tank without showing any pattern consistent with negative phototaxis.
(TIF)
