# Supplementary figures and images for: Host-Seeking Behavior and Dispersal of Triatoma infestans, a Vector of Chagas Disease, under Semi-field Conditions
Source: PLoS Negl Trop Dis. 2015 Jan 8;9(1):e3433. doi: 10.1371/journal.pntd.0003433 (PMC4287539; doi:10.1371/journal.pntd.0003433)

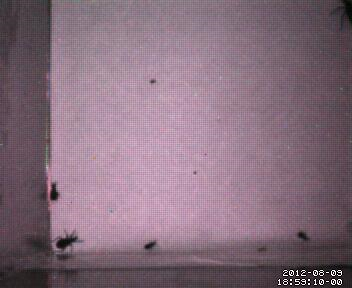

Supplement: S1 Fig — In our pilot study triatomine insects under white light tend to stay on the borders of the tank and only move along the borders, a behavior consistent with negative phototaxis. (TIF) [file pntd.0003433.s002.tif]

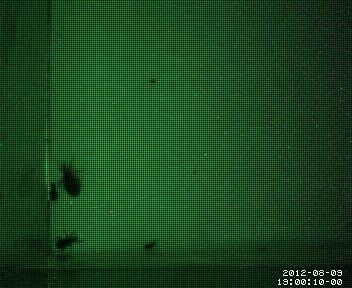

Supplement: S2 Fig — In our pilot study triatomine insects under green light tend to stay on the borders of the tank and only move along the borders, a behavior consistent with negative phototaxis. (TIF) [file pntd.0003433.s003.tif]

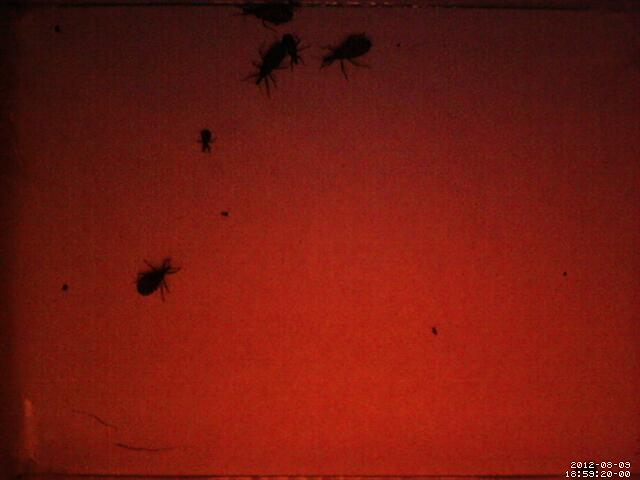

Supplement: S3 Fig — In our pilot study triatomine insects under red light move all over the tank without showing any pattern consistent with negative phototaxis. (TIF) [file pntd.0003433.s004.tif]
